# Health of migrants, refugees and asylum seekers in detention in Tripoli, Libya, 2018-2019: Retrospective analysis of routine medical programme data

**DOI:** 10.1371/journal.pone.0252460

**Published:** 2021-06-04

**Authors:** Anna Kuehne, Elburg van Boetzelaer, Prince Alfani, Adolphe Fotso, Hitham Elhammali, Tom Khamala, Trygve Thorson, Ilina Angelova, Bianca Benvenuti, Biserka Pop-Stefanija, Maria Verdecchia, Ronald Kremer

**Affiliations:** 1 Médecins Sans Frontières, Berlin, Germany; 2 Médecins Sans Frontières, London, United Kingdom; 3 Médecins Sans Frontières, Amsterdam, The Netherlands; 4 Médecins Sans Frontières, Tripoli, Libya; The University of Georgia, UNITED STATES

## Abstract

Libya is a major transit and destination country for international migration. UN agencies estimates 571,464 migrants, refugees and asylum seekers in Libya in 2021; among these, 3,934 people are held in detention. We aimed to describe morbidities and water, hygiene, and sanitation (WHS) conditions in detention in Tripoli, Libya. We conducted a retrospective analysis of data collected between July 2018 and December 2019, as part of routine monitoring within an Médecins Sans Frontières (MSF) project providing healthcare and WHS support for migrants, refugees and asylum seekers in some of the official detention centres (DC) in Tripoli. MSF had access to 1,630 detainees in eight different DCs on average per month. Only one DC was accessible to MSF every single month. The size of wall openings permitting cell ventilation failed to meet minimum standards in all DCs. Minimum standards for floor space, availability of water, toilets and showers were frequently not met. The most frequent diseases were acute respiratory tract infections (26.9%; 6,775/25,135), musculoskeletal diseases (24.1%; 6,058/25,135), skin diseases (14.1%; 3,538/25,135) and heartburn and reflux (10.0%; 2,502/25,135). Additionally, MSF recorded 190 cases of violence-induced wounds and 55 cases of sexual and gender-based violence. During an exhaustive nutrition screening in one DC, linear regression showed a reduction in mid-upper arm circumference (MUAC) of 2.5mm per month in detention (95%-CI 1.3–3.7, p<0.001). Detention of men, women and children continues to take place in Tripoli. Living conditions failed to meet minimum requirements. Health problems diagnosed at MSF consultations reflect the living conditions and consist largely of diseases related to overcrowding, lack of water and ventilation, and poor diet. Furthermore, every month that people stay in detention increases their risk of malnutrition. The documented living conditions and health problems call for an end of detention and better protection of migrants, refugees and asylum seekers in Libya.

## Introduction

*“I was working a day to day job for one month until one day I was taken by some police officer ‘cause I was illegal in the country*. *I stayed in detention for one month*, *January 2019*. *The detention called [name of a detention centre in Tripoli]*. *The situation was really bad*. *The guards use to beat us every day for no reason*, *just out of boredom*. *There is no dignity…*.*”* Account told by a 16-year-old Sudanese boy to Médecins Sans Frontières (MSF) staff, after being rescued on the Mediterranean Sea in August 2019.

Libya is geographically the fourth largest country in Africa, with a population of 6.7 million [1]. Since 2011, and the ousting of Colonel Muammar Gaddafi, successive Libyan governments have struggled to assert control amid the proliferation of rival political groups competing for power [2]. This has resulted in wide-scale conflict and system collapse with dire security, social, and economic consequences. The conflict is indiscriminate in nature and attacks on civilians and infrastructure are frequent; the conflict has resulted in substantial internal population displacement [2]. Violent clashes between armed groups escalated again in April 2019 and occurred throughout the year in Tripoli [2]. According to the Human Development Index, Libya has fallen from a ranking of 67 to 110, and the economic crisis is affecting all parts of society [2, 3], with migrants and refugees considered some of the most vulnerable groups in the country [2].

Libya is a major transit and destination country for international migration [4]. While the majority of migrants in Libya report that they originally intended to stay and work in Libya, the country has become an important departure point for migrants to Europe [4, 5]. As of February 2021, there were 571,464 migrants, refugees and asylum seekers in Libya [6]. In response to pressure from European countries, Libya implemented restrictive policies to stem the transit of people on the move [7, 8]. The European Union (EU), on its side, set the aim to rapidly stem the flow of irregular arrivals in Europe and prevent departures from Libya as one of the pillars of its migration policy for the Central Mediterranean [9, 10]. Since 2017, the EU has funded a project of the Italian government to support migration management in Libya [11]. Capacitating the Libyan Coast Guards to assume the responsibility in an expanded search and rescue region has been one of the central components of the project [11–13]. Improving the human rights situation for migrants has been a cross-cutting objective [12, 13]. However, despite the publicly available evidence of continued abuses against migrants and refugees in Libya [7, 14–18], neither the European Commission nor the Italian government have to date disclosed information on human rights performance monitoring.

Since 2017, more than 50,000 people have been returned to Libya by the Libyan Coast Guards [19–23]. This has been accepted as the new normal and, as departures from Libya and arrivals in Europe have decreased, the EU has publicly praised the efforts of the Libyan Coast Guards [24]. Across Libya, there are numerous detention centres (DCs) run by authorities or a variety of militia or traffickers [7, 8, 25]. Migrants, refugees and asylum seekers are frequently intercepted on the Mediterranean Sea, or are arrested in neighbourhood raids, and are subsequently held in DCs. The United Nations High Commissioner for Refugees estimates that 3,934 migrants, refugees and asylum seekers are still held in official DCs in Libya, as of February 2021 [26]. An unidentified number of people are additionally held in unofficial places of captivity. DCs are officially managed by the Libyan Directorate Combatting Illegal Migration (DCIM), which is also one of the target beneficiaries of EU funding [12, 25, 27]. The true number of detention centres and detainees in Libya is unknown.

DCs are consisting of hangars, regularly near or within the vicinity of military or armed group facilities. Conditions in official DCs are often reported to be grossly inadequate, featuring chronic overcrowding, poor sanitation, insufficient access to healthcare, and inadequate quantity and quality of food [7, 17]. Cells are usually large and overcrowded, often with tiny windows, only one door and metal roofs that contribute to substantial heat in summer months and cold in winter. Individuals in detention in Libya have reported forced labour, violence, rape, abuse and torture in DCs [7, 17]. Several organisations have documented that detention centres are often run by, or heavily influenced by militias controlling the surrounding territory, often seeking to gain financially from their activities [7, 8, 17]. Migrants, refugees and asylum seekers typically have no access to judicial processes and effective monitoring systems do not exist, thus there is no oversight of guards or protection against violations and abuse [7, 17, 25].

MSF is present in Libya and the Central Mediterranean to respond to loss of life and the abuse and neglect suffered by migrants, refugees and asylum seekers, through the provision of lifesaving interventions, medical care, protection measures and advocating for changes to harmful migration deterrence policies and practices [28–30]. MSF has advocated for the end of arbitrary detention in Libya for several years and provided medical care within several of Tripoli’s DC’s since June 2016. MSF typically aims to visit the population of each DC at least once each week to provide general medical, obstetrical and gynaecological consultations, including antenatal and postnatal care (ANC and PNC), and mental health consultations. Patients that require further health care, such as surgical or inpatient care, are referred to clinics outside DCs. Furthermore, MSF’s water, hygiene and sanitation (WHS) team provides and treats water in some DCs, improving the existing latrines and showers and implementing vector control activities for communicable diseases such as scabies. Additionally, MSF distributes cleaning material and other non-food items (underwear, hygiene articles etc.) to detainees.

To date, no epidemiological information on health of migrants, refugees and asylum seekers in detention in Tripoli is available. We aimed to contribute to the systematic epidemiological description of health conditions among detained migrants, refugees and asylum seekers in Libya. Our analysis used a retrospective analysis of routine medical and WHS data, in order to contribute to documentation of living conditions and health in Libya’s DCs.

## Methods

We conducted a retrospective analysis of data collected between July 2018 and December 2019 as part of the routine monitoring of an MSF project that provides health care and WHS services for migrants, refugees and asylum seekers in detention in Tripoli, Libya.

### Study population

The study population within our analysis consisted of all migrants, refugees and asylum seekers to which MSF had access during visits to DCs in Tripoli, Libya from July 2018 to December 2019.

### Data sources and data collection

Routine programme data covered the total number of detainees seen during every mobile clinic visit by the medical team; this was recorded in a database every month for the last visit of the month, in all DCs to which MSF had access.

Routine environmental data was routinely collected once per month by the MSF WHS specialist in each DC, documenting WHS conditions, with comparisons made against standards for prisons [31] and humanitarian programmes [32]. These standards include those for floor space (m^2^/person), ventilation (size of wall opening allowing ventilation/cell) and water, shower and toilet availability (persons/tap or shower or toilet).

For routine medical data, data collection took place as a routine medical activity. The datasets used in our analysis contained data from all detainees presenting at MSF outpatient consultations with a medical complaint or that were referred to a medical facility, in all DCs to which MSF had access. Data collection within outpatient clinics includes the number of new and follow-up outpatient department (OPD) consultations, the number of sexual and reproductive health consultations, the number of sexual and gender-based violence consultations, the number of tuberculosis (TB) patients and the number of referrals for TB and other conditions that could not be treated in DCs. For OPD consultations, the presenting main medical condition is documented on first visit. Suspect TB patients that were identified during OPD consultations based on clinical symptoms were referred for diagnostic procedures to an outside private clinic. If TB was confirmed, patients were treated and followed-up by MSF. There was no TB screening in place.

For nutrition screening data, these outcomes pertain to an exhaustive nutrition screening project that was conducted in one DC in January 2019 by MSF following reports of lack of food and cases of severe malnutrition. The assessment carried out by MSF staff included all detainees within that DC at the time, recording age, sex, self-reported duration of stay in the DC, middle upper arm circumference (MUAC) and body mass index (BMI) in kg/(height in m)^2^.

### Definitions

We defined MSF OPD consultations as all MSF OPD consultations in a given time, including consultations for children aged under five years, and any individuals aged five years and over, with both new and follow-up consultations included, unless specified otherwise.

For malnutrition, primary adult malnutrition as detected in OPD consultations was defined as a MUAC<160mm. Adult malnutrition as detected during nutrition screening in one DC was defined based on BMI cut-offs: <16, severe; 16-<17, moderate; 17-<18.5, mild; and ≥18.5 normal [33].

We defined “access to DCs” as meaning that consultations could be conducted as originally intended. “Partial access to DCs” was defined as access being denied, either to certain detainees or at specific dates where visits were intended.

### Data analysis

Following data cleaning and transfer to STATA version 15 (Stata Corporation, Texas, USA), we conducted descriptive analysis of the available programme, medical, and environmental data.

For the analysis of malnutrition data, we excluded cases with incomplete or inconclusive data on length of stay and malnutrition, and cases that were aged under 18 years.

Indicators were either calculated as proportions (e.g. morbidities, malnutrition) or medians with their range (e.g. age, lengths of stay, living space in m^2^ per person). We conducted linear regression to analyse the impact of length of stay on malnutrition and plotted residual to check for patterns. All results are presented in text, tables or graphs as appropriate.

### Ethical considerations

This was a retrospective analysis of routinely collected data; therefore, it was exempted from full ethical review by the MSF research committee. The data in the utilized datasets did not contain individual identifiers; it was password protected and only accessible by the principal investigator.

## Results

### Access to migrants, refugees and asylum seekers in detention

Over the course of 18 months, MSF had access to 29,346 detainees according to monthly counts, including 2,942 women (10.0%; 2,944/29,346) and 1,324 minors (4.5%; 1,324/29,346).

**Fig 1:**
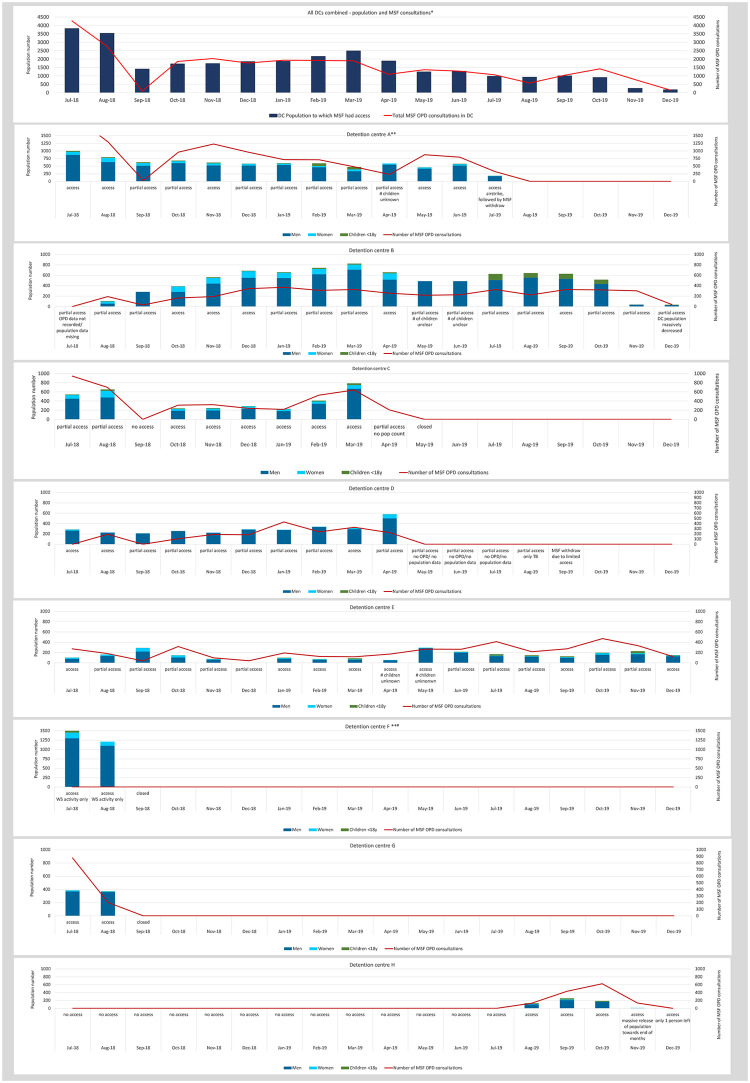
Health of migrants, refugees and asylum seekers in detention in Tripoli, Libya 2018–2019: Number of population and MSF outpatient consultations in 8 detention centres by month. DC: Detention centre; OPD: outpatient department. * Scale different to other graphs; for this graph the maximum is 4,500 population and MSF consultations. ** Scale different to other graphs; for this graph the maximum is 1,500 population and MSF consultations. ^#^ MSF Water, hygiene and sanitation activities only.

From July 2018 to December 2019, MSF had access to eight different DCs in Tripoli. MSF provided patient care and WHS support in seven DCs. In one DC, MSF provided WHS support only and no medical care. Access was negotiated week by week and only one DC could be visited every month during the time period covered by our analysis (Fig 1).

### Living conditions for migrants, refugees and asylum seekers in detention

The median number of persons per drinking water tap did not meet minimum standards [31, 32] for male detainees’ cells in four of seven DCs with documented WHS conditions (Table 1).

**Table 1:** Health of migrants, refugees and asylum seekers in detention in Tripoli, Libya 2018–2019: Median water, hygiene and sanitation indicators for access to water, toilets and showers and space and ventilation available in 8 detention centres.

	DC A			DC B			DC C			DC D			DC E			DC F			DC G			DC H		
Water, hygiene, sanitation (WHS) activities																								
# months with MSF activities	13			18			9			14			18			2			2			5		
# months with WHS activities	13			16			8			12			14			2			2			5		
# months with data collection	10			13			8			9			6			2			2			0		
	median	min	max	median	min	max	median	min	max	median	min	max	median	min	max	median	min	max	median	min	max	median	min	max
Drinking water–standard*: max 50 people per tap																								
Male/tap	29	18	30	100	50	147	27	10	37	69	0	95	27	12	50	200	183	217	182.5	180	185			
Female/tap	14	12	23				9	9	44	26	26	26	12	6	24	39	28	50						
Washing water–standard*: max 50 people per tap																								
Male/tap	29	25	41	100	50	110	10.5	10	27	76	55	95	15	8	28	120	110	130	33.5	33	34			
Female/tap	14	14	27	25	21	25	9	8	44	26	26	26	6.5	3	12	22.5	16	29	5.5	5	6			
Hygiene and sanitation–standard*: max 50 people per toilet and shower																								
Male/toilet	39	38	73	71	37	110	9.5	6	32	35	22	133	56	31	111	150.5	138	163	73	72	74			
Female/toilet	30	28	41	33.5	25	50	20.5	13	88	1	1	28	8	6	24	39	28	50	4.5	4	5			
Male/shower	39	38	73	71	37	176	18.5	7	32	22	22	133	56	31	111	150.5	138	163	73	72	74			
Female/shower	24	21	41	50	42	50	21	13	88	14.5	1	28	8	6	24	39	28	50	16	14	18			
Space–standard*: 3.4 m² floor area per person absolute minimum in dormitories																								
Male—m2/person	3.3	2	3.4	1.25	0.1	3.8	9.4	3.8	9.7	3	0.8	3.3	1.5	0.7	2	1.5	1.4	1.6	2.25	2.2	2.3			
Female—m2/person	4.2	2.4	4.8	6.7	6.7	7.9	4.3	0.9	4.4	6.7	5	240	1.2	0.7	2.2	2.2	1.6	2.8	3.05	2.7	3.4			
Ventilation of cells–standard*: minimum size of the cell opening ≥ 10% total floor space																								
Male cell opening in % of the floor area	1.4	1	1.4	4	4	5	0.7	0.7	0.7	2	1.3	2	2.5	2.5	2.5	3	3	3	2	2	2			
Female cell opening in % of the floor area	2.8	2.8	3	4	4	4	2.7	2.6	2.7	3	3	3	4	4	4	8	8	8	2.1	1.2	3			

DC: detention centre; WHS: Water Hygiene Sanitation.

*Minimum standards as defined by SPHERE and the International Committee of the Red Cross [31, 32]

The median space (in m^2^) required per person met the minimum standards [31, 32] in only one DC for male cells, and in four DCs for cells intended for female detainees. The median ventilation (measured as the cell opening as a percentage of the floor area) failed to meet minimum standards in all DCs [31, 32].

### Health and healthcare in detention

Between July 2018 and December 2019, MSF conducted 27,307 OPD consultation in total; among these, 1,979 were follow-up consultations (7.2%, 1,979/27,307) for a fluctuating population of approximately 29,346 detainees (Table 2).

**Table 2:** Health of migrants, refugees and asylum seekers in detention in Tripoli, Libya 2018–2019: MSF consultations and referrals in 7 detention centres by month.

	Jul-18	Aug-18	Sep-18	Oct-18	Nov-18	Dec-18	Jan-19	Feb-19	Mar-19	Apr-19	May-19	Jun-19	Jul-19	Aug-19	Sep-19	Oct-19	Nov-19	Dec-19	Total
DC population estimate	3826	3537	1415	1721	1736	1854	1875	2166	2497	1891	1250	1285	986	926	1014	911	268	188	*
**Outpatient department (OPD)**																			
MSF OPD consultations new	4127	2627	95	1829	1945	1646	1783	1736	1702	993	1220	1137	944	512	941	1234	709	148	**25328**
MSF OPD consultations follow-up	143	135	0	36	86	119	146	182	196	99	142	150	120	63	96	184	69	13	**1979**
MSF OPD consultations total	4270	2762	95	1865	2031	1765	1929	1918	1898	1092	1362	1287	1064	575	1037	1418	778	161	**27307**
**Sexual and reproductive health (SRH) and sexual and gender-based violence (SGBV)**																			
MSF SRH consultations total	119	105	0	157	104	69	100	76	82	56	27	30	1	5	3	6	12	1	**953**
MSF 1st ANC visits	19	28	0	14	13	15	6	9	9	9	9	4	0	4	1	3	1	0	**144**
MSF ANC follow-up visits	55	61	0	86	50	21	41	32	51	29	17	22	0	0	2	0	10	1	**478**
MSF PNC visits	41	10	0	55	41	31	52	32	21	18	1	0	1	0	0	1	1	0	**305**
MSF other obstetric and gynecological visits	4	6	0	2	0	2	1	3	1	0	0	4	0	1	0	2	0	0	**26**
MSF deliveries after referral	4	4	1	3	5	1	2	0	4	0	0	1	0	0	0	1	2	0	**28**
MSF SGBV consultations	4	6	4	7	7	3	1	4	4	1	6	4	1	2	1	0	0	0	**55**
**Tuberculosis (TB)**																			
MSF newly confirmed TB cases	17	13	3	24	22	8	11	14	12	13	18	7	11	8	8	-	1	0	**190**
MSF total TB cases in cohort	79	90	69	75	91	92	91	80	84	54	55	53	48	47	43	-	13	13	*
**Referrals to clinics outside of DC**																			
MSF referrals to IPD	131	63	32	78	41	30	33	42	49	46	35	43	35	20	21	28	9	1	**737**
MSF referrals to OPD	18	28	18	36	42	21	27	30	46	35	31	30	38	19	27	27	23	2	**498**
Total MSF referrals	149	91	50	114	83	51	60	72	95	81	66	73	73	39	48	55	32	3	**1235**

DC: Detention centre; OPD: outpatient department; SRH: Sexual and reproductive health; SGBV: Sexual and gender-based violence; ANC: Antenatal care; PNC: Postnatal care; TB: Tuberculosis; IPD: inpatient department.

*Individuals may have been double counted in successive months, it is therefore it is not possible to calculate a total

MSF performed 144 first ANC consultations (4.9% (144/2,944) of the female population had a first ANC visit) and conducted 953 sexual and reproductive health (SRH) consultations overall (Table 2). Of the female DC population, 32.4% was seen for a SRH problem, including pregnancy care (953/2,942). Additionally, 55 cases of sexual violence reported as occurring within the DC, or outside, were documented (Table 2). MSF identified 190 cases of TB—among them two cases of multi-drug resistant TB—and followed up 13 to 92 cases of TB in any given month (Table 2). MSF organised 1,235 medical referrals to non-MSF clinics outside of the DCs (4.2% of the DC population; 1,235/29,346) (Table 2). Of the referred patients, 59.7% (737/1,235) were referred for inpatient treatment to the hospital. There is no complete list of diagnosis at referral; examples of common reasons for referrals were physical trauma–both, violence-related such as gunshots, stab wounds and beatings, as well as incidental trauma caused by falling, crushes sustained on overcrowded boats or chemical burns derived from fuel. Other common reasons for referral were pregnancy check-ups and pregnancy complications or labour, TB manifestations referred for diagnostics, psychiatric illnesses including post traumatic distress and untreated chronic non-communicable diseases such as diabetes.

Among children aged under five years, MSF conducted 193 consultations for new diseases, 10 follow-up consultations and 42 dressings or injections. Consultations among children aged under five accounted for 0.78% (213/27,307) of all consultations. Among all new consultations, 44.6% (86/193) were related to respiratory tract infections, and 25.4% (49/193) were skin diseases, including scabies (30.6% of all skin diseases; 15/49) (Table 3).

**Table 3:** Health of migrants, refugees and asylum seekers in detention in Tripoli, Libya 2018–2019: MSF outpatient department consultations among children < 5 years in 7 detention centres by month.

Diagnosis in outpatient department (OPD) in children < 5 years of age																			
	Jul-18	Aug-18	Sep-18	Oct-18	Nov-18	Dec-18	Jan-19	Feb-19	Mar-19	Apr-19	May-19	Jun-19	Jul-19	Aug-19	Sep-19	Oct-19	Nov-19	Dec-19	Total
Acute upper respiratory tract infection	10	11	0	6	8	7	9	1	5	9	2	5	1	0	0	3	0	0	**77**
Acute lower respiratory tract infection	0	1	0	0	2	2	2	0	0	1	1	0	0	0	0	0	0	0	**9**
Skin disease	2	9	0	9	1	2	1	2	6	4	0	5	7	0	1	0	0	0	**49**
Acute watery diarrhoea	2	1	0	0	0	0	1	1	2	0	0	0	0	0	0	0	0	0	**7**
Fever of unknown origin	3	0	0	0	0	2	0	0	0	0	0	0	0	0	0	0	0	0	**5**
Dental problem	1	1	0	2	0	0	0	0	0	0	0	1	0	0	0	0	0	0	**5**
Non-violence related injury	0	0	0	2	0	1	0	0	1	0	0	0	0	0	0	0	0	0	**4**
Heartburn/ reflux	3	0	0	0	0	0	1	0	0	0	0	0	0	0	0	0	0	0	**4**
Eye infection	2	1	0	0	0	0	0	0	0	0	0	0	0	0	0	0	0	0	**3**
Confirmed malaria	2	0	0	0	0	0	0	0	0	0	0	0	0	0	0	0	0	0	**2**
Dehydration	2	0	0	0	0	0	0	0	0	0	0	0	0	0	0	0	0	0	**2**
Urinary tract infection	0	1	0	0	0	0	0	0	0	0	0	0	0	0	0	0	0	0	**1**
Chronic diseases	0	0	0	0	0	0	0	0	1	0	0	0	0	0	0	0	0	0	**1**
Fuel burn	1	0	0	0	0	0	0	0	0	0	0	0	0	0	0	0	0	0	**1**
Severe acute malnutrition	0	0	0	0	0	0	0	0	0	0	0	0	0	0	0	0	0	0	**0**
Other	3	0	0	0	0	0	0	2	0	1	17	0	0	0	0	0	0	0	**23**
**Total**	**31**	**25**	**0**	**19**	**11**	**14**	**14**	**6**	**15**	**15**	**20**	**11**	**8**	**0**	**1**	**3**	**0**	**0**	**193**

OPD: Outpatient department

Among children aged 5 years and older and adults, MSF conducted 25,135 consultations for new diseases, 1,969 follow-up consultations and 939 dressings or injections (Table 4).

**Table 4:** Health of migrants, refugees and asylum seekers in detention in Tripoli, Libya 2018–2019: MSF outpatient department consultations among patients ≥ 5 years and adults in 7 detention centres by month.

**Diagnosis in outpatient department (OPD) in patients ≥ 5 years of age**																				
	Jul-18		Aug-18		Sep-18		Oct-18		Nov-18		Dec-18		Jan-19		Feb-19		Mar-19		Apr-19	
	n	%	n	%	n	%	n	%	n	%	n	%	n	%	n	%	n	%	n	%
Headache/ musculoskeletal pain	1035	25.3%	643	24.7%	21	22.1%	412	22.8%	462	23.9%	399	24.4%	392	22.2%	392	22.7%	370	21.9%	219	22.4%
Acute upper respiratory tract infection	817	19.9%	482	18.5%	11	11.6%	354	19.6%	415	21.5%	313	19.2%	339	19.2%	342	19.8%	287	17.0%	172	17.6%
Acute lower respiratory tract infection	376	9.2%	241	9.3%	9	9.5%	216	11.9%	186	9.6%	121	7.4%	228	12.9%	204	11.8%	192	11.4%	90	9.2%
Skin disease	581	14.2%	353	13.6%	15	15.8%	240	13.3%	254	13.1%	200	12.3%	208	11.8%	270	15.6%	283	16.8%	156	16.0%
Heartburn/ reflux	476	11.6%	332	12.8%	9	9.5%	186	10.3%	213	11.0%	196	12.0%	172	9.7%	125	7.2%	141	8.4%	84	8.6%
Constipation	23	0.6%	9	0.3%	0	0.0%	16	0.9%	39	2.0%	48	2.9%	38	2.1%	35	2.0%	24	1.4%	16	1.6%
Acute watery diarrhoea	92	2.2%	134	5.1%	3	3.2%	38	2.1%	42	2.2%	40	2.5%	25	1.4%	42	2.4%	13	0.8%	16	1.6%
Acute bloody diarrhoea	7	0.2%	0	0.0%	1	1.1%	1	0.1%	2	0.1%	0	0.0%	0	0.0%	0	0.0%	0	0.0%	0	0.0%
Dental problem	175	4.3%	94	3.6%	3	3.2%	107	5.9%	87	4.5%	70	4.3%	67	3.8%	57	3.3%	48	2.8%	53	5.4%
Chronic diseases	24	0.6%	20	0.8%	0	0.0%	11	0.6%	11	0.6%	32	2.0%	56	3.2%	71	4.1%	74	4.4%	25	2.6%
Urinary tract infection	71	1.7%	43	1.7%	4	4.2%	60	3.3%	46	2.4%	42	2.6%	48	2.7%	23	1.3%	25	1.5%	21	2.1%
Eye infection	56	1.4%	56	2.2%	2	2.1%	32	1.8%	41	2.1%	35	2.1%	44	2.5%	35	2.0%	26	1.5%	18	1.8%
Dehydration	91	2.2%	55	2.1%	1	1.1%	2	0.1%	0	0.0%	9	0.6%	10	0.6%	10	0.6%	13	0.8%	3	0.3%
Anaemia	37	0.9%	39	1.5%	3	3.2%	38	2.1%	19	1.0%	19	1.2%	4	0.2%	15	0.9%	7	0.4%	13	1.3%
Gynaecological	37	0.9%	24	0.9%	11	11.6%	26	1.4%	25	1.3%	14	0.9%	13	0.7%	7	0.4%	24	1.4%	2	0.2%
Violence-related trauma, wound, burn	21	0.5%	10	0.4%	0	0.0%	7	0.4%	9	0.5%	10	0.6%	7	0.4%	10	0.6%	5	0.3%	5	0.5%
Non-violence related injury	32	0.8%	11	0.4%	0	0.0%	4	0.2%	12	0.6%	11	0.7%	5	0.3%	6	0.3%	10	0.6%	9	0.9%
Tuberculosis (suspected)	9	0.2%	2	0.1%	0	0.0%	7	0.4%	5	0.3%	7	0.4%	20	1.1%	1	0.1%	0	0.0%	3	0.3%
Fever of unknown origin	19	0.5%	10	0.4%	0	0.0%	4	0.2%	10	0.5%	6	0.4%	5	0.3%	6	0.3%	6	0.4%	7	0.7%
Fuel burn	29	0.7%	0	0.0%	0	0.0%	0	0.0%	1	0.1%	0	0.0%	1	0.1%	0	0.0%	15	0.9%	0	0.0%
Severe acute malnutrition	2	0.0%	1	0.0%	2	2.1%	1	0.1%	10	0.5%	2	0.1%	2	0.1%	1	0.1%	4	0.2%	4	0.4%
Pregnancy related consultation	0	0.0%	6	0.2%	0	0.0%	4	0.2%	0	0.0%	0	0.0%	6	0.3%	1	0.1%	0	0.0%	1	0.1%
Sexually transmitted infection—male	0	0.0%	1	0.0%	0	0.0%	3	0.2%	2	0.1%	2	0.1%	1	0.1%	0	0.0%	0	0.0%	1	0.1%
Sexually transmitted infection—female	0	0.0%	0	0.0%	0	0.0%	2	0.1%	1	0.1%	0	0.0%	1	0.1%	0	0.0%	0	0.0%	2	0.2%
Common Psychiatric Disorders	1	0.0%	0	0.0%	0	0.0%	1	0.1%	0	0.0%	0	0.0%	4	0.2%	2	0.1%	1	0.1%	1	0.1%
Severe Psychiatric Disorders	0	0.0%	1	0.0%	0	0.0%	0	0.0%	0	0.0%	2	0.1%	0	0.0%	0	0.0%	1	0.1%	0	0.0%
Confirmed malaria	0	0.0%	0	0.0%	0	0.0%	0	0.0%	0	0.0%	0	0.0%	0	0.0%	2	0.1%	0	0.0%	0	0.0%
Other	85	2.1%	35	1.3%	0	0.0%	38	2.1%	42	2.2%	54	3.3%	73	4.1%	73	4.2%	118	7.0%	57	5.8%
**Total**	**4096**		**2602**		**95**		**1810**		**1934**		**1632**		**1769**		**1730**		**1687**		**978**	
Follow-up consultations	143		135		0		31		86		119		146		177		196		99	
Dressing/injection room	152		100		0		77		98		82		47		39		76		16	
**Diagnosis in outpatient department (OPD) in patients ≥ 5 years of age**																				
	May-19		Jun-19		Jul-19		Aug-19		Sep-19		Oct-19		Nov-19		Dec-19		Total			
	n	%	n	%	n	%	n	%	n	%	n	%	n	%	n	%	**n**	**%**		
Headache/ musculoskeletal pain	294	24.5%	282	25.0%	248	26.5%	157	30.7%	250	26.6%	273	22.2%	175	24.7%	34	23.0%	**6058**	**24.1%**		
Acute upper respiratory tract infection	157	13.1%	196	17.4%	137	14.6%	79	15.4%	105	11.2%	147	11.9%	112	15.8%	29	19.6%	**4494**	**17.9%**		
Acute lower respiratory tract infection	82	6.8%	46	4.1%	53	5.7%	33	6.4%	30	3.2%	115	9.3%	46	6.5%	13	8.8%	**2281**	**9.1%**		
Skin disease	219	18.3%	136	12.1%	107	11.4%	70	13.7%	127	13.5%	161	13.1%	140	19.7%	18	12.2%	**3538**	**14.1%**		
Heartburn/ reflux	132	11.0%	114	10.1%	88	9.4%	26	5.1%	63	6.7%	96	7.8%	36	5.1%	13	8.8%	**2502**	**10.0%**		
Constipation	26	2.2%	31	2.8%	27	2.9%	15	2.9%	37	3.9%	58	4.7%	52	7.3%	7	4.7%	**501**	**2.0%**		
Acute watery diarrhoea	28	2.3%	10	0.9%	17	1.8%	2	0.4%	6	0.6%	18	1.5%	4	0.6%	6	4.1%	**536**	**2.1%**		
Acute bloody diarrhoea	0	0.0%	0	0.0%	1	0.1%	0	0.0%	0	0.0%	2	0.2%	0	0.0%	0	0.0%	**14**	**0.1%**		
Dental problem	42	3.5%	36	3.2%	35	3.7%	24	4.7%	67	7.1%	68	5.5%	34	4.8%	9	6.1%	**1076**	**4.3%**		
Chronic diseases	47	3.9%	50	4.4%	47	5.0%	20	3.9%	35	3.7%	65	5.3%	20	2.8%	3	2.0%	**611**	**2.4%**		
Urinary tract infection	23	1.9%	49	4.4%	36	3.8%	15	2.9%	43	4.6%	26	2.1%	12	1.7%	5	3.4%	**592**	**2.4%**		
Eye infection	26	2.2%	26	2.3%	22	2.4%	8	1.6%	23	2.4%	21	1.7%	4	0.6%	1	0.7%	**476**	**1.9%**		
Dehydration	1	0.1%	14	1.2%	17	1.8%	8	1.6%	16	1.7%	20	1.6%	4	0.6%	0	0.0%	**274**	**1.1%**		
Anaemia	7	0.6%	6	0.5%	4	0.4%	2	0.4%	3	0.3%	4	0.3%	3	0.4%	0	0.0%	**223**	**0.9%**		
Gynaecological	6	0.5%	5	0.4%	3	0.3%	2	0.4%	9	1.0%	13	1.1%	0	0.0%	2	1.4%	**223**	**0.9%**		
Violence-related trauma, wound, burn	20	1.7%	31	2.8%	7	0.7%	3	0.6%	15	1.6%	22	1.8%	7	1.0%	1	0.7%	**190**	**0.8%**		
Non-violence related injury	6	0.5%	7	0.6%	10	1.1%	6	1.2%	4	0.4%	4	0.3%	5	0.7%	0	0.0%	**142**	**0.6%**		
Tuberculosis (suspected)	1	0.1%	0	0.0%	11	1.2%	7	1.4%	11	1.2%	10	0.8%	8	1.1%	0	0.0%	**102**	**0.4%**		
Fever of unknown origin	5	0.4%	1	0.1%	1	0.1%	3	0.6%	2	0.2%	0	0.0%	0	0.0%	0	0.0%	**85**	**0.3%**		
Fuel burn	2	0.2%	18	1.6%	0	0.0%	1	0.2%	2	0.2%	1	0.1%	0	0.0%	0	0.0%	**70**	**0.3%**		
Severe acute malnutrition	3	0.3%	0	0.0%	1	0.1%	0	0.0%	1	0.1%	0	0.0%	0	0.0%	0	0.0%	**34**	**0.1%**		
Pregnancy related consultation	0	0.0%	0	0.0%	0	0.0%	0	0.0%	5	0.5%	1	0.1%	0	0.0%	0	0.0%	**24**	**0.1%**		
Sexually transmitted infection—male	0	0.0%	0	0.0%	0	0.0%	1	0.2%	0	0.0%	0	0.0%	0	0.0%	0	0.0%	**11**	**0.0%**		
Sexually transmitted infection—female	0	0.0%	2	0.2%	0	0.0%	0	0.0%	1	0.1%	4	0.3%	0	0.0%	0	0.0%	**13**	**0.1%**		
Common Psychiatric Disorders	1	0.1%	0	0.0%	0	0.0%	1	0.2%	0	0.0%	0	0.0%	0	0.0%	0	0.0%	**12**	**0.0%**		
Severe Psychiatric Disorders	1	0.1%	0	0.0%	0	0.0%	1	0.2%	0	0.0%	1	0.1%	0	0.0%	0	0.0%	**7**	**0.0%**		
Confirmed malaria	0	0.0%	0	0.0%	0	0.0%	0	0.0%	0	0.0%	0	0.0%	0	0.0%	0	0.0%	**2**	**0.0%**		
Other	71	5.9%	66	5.9%	64	6.8%	28	5.5%	85	9.0%	96	7.8%	47	6.6%	7	4.7%	**1039**	**4.1%**		
**Total**	**1200**		**1126**		**936**		**512**		**940**		**1231**		**709**		**148**		**25135**	**100%**		
Follow-up consultations	142		150		120		63		96		184		69		13		**1969**			
Dressing/injection room	37		67		43		16		41		39		6		3		**939**			

OPD: Outpatient department

The number of monthly OPD consultations for new diseases varied from 4,096 in July 2018 to 148 in December 2019 (Table 4). Consultations approximately halved between August July and October 2018 due to ongoing conflict in Tripoli, and halved again between March and April 2019, due to intensified fighting in Tripoli.

The most frequently diagnosed new conditions were acute respiratory tract infections (27%; 6,775/25,135), musculoskeletal diseases (24.1%; 6,058/25,135), skin diseases (14.1%; 3,538/25,135) and heartburn and reflux (10.0%; 2,502/25,135) (Table 4). Among the diagnosis of skin diseases, 41.9% were scabies (1,483/3,538).

### Malnutrition in detention

Over the 18-month period, MSF detected 34 cases of primary adult malnutrition in DCs during OPD consultations, defined as a MUAC<160mm. MSF conducted extensive nutrition screening in DC “D” in January 2019, including assessments done amongst all 302 detainees at the time. All detainees were male, and the mean age of detainees was 23 years (median 22, range 10–46); average self-reported duration of stay in the DC at the point of screening was 4.8 months (median 6, range 1–12). Mean MUAC among detainees ≥ 18 years (n = 233) was 264.5mm (median 262, range 208–364). Mean BMI among detainees ≥ 18 years was 21.1 kg/m^2^ (median 20.7, range 16.1–37.7). Among all detainees aged above 18 years, we found 3.9% (9/233) with moderate malnutrition and 12.0% (28/233) with mild malnutrition.

Using linear regression analysis, we assessed the association between self-reported duration of stay and detainees’ MUAC measurement. We estimated a reduction in mean MUAC of 2.5mm per month of length of stay (95% CI 1.3–3.7, p<0.001), after adjusting for detainees’ age.

Additionally, we assessed the association between self-reported duration of stay and detainees’ BMI. We estimated a reduction in mean BMI of 0.16 kg/m^2^ per month of length of stay (95% CI: 0.15–0.29, p = 0.028) after adjusting for detainees’ age.

## Discussion

At any given time between July 2018 and December 2019 there were between 188 and 3,826 migrants, refugees and asylum seekers in eight DCs in Tripoli to which MSF had access. Even after the beginning of the conflict in Tripoli in April 2019 between the Libyan Government of National Accord and the Libyan National Army, more than 1,800 migrants, refugees and asylum seekers, including women and children, remained in DCs near conflict-affected areas. The number of migrants, refugees and asylum seekers decreased to fewer than 900 detainees for the first time in November 2019. On 2 July 2019, one of the DCs within which MSF was providing healthcare was hit by an airstrike, killing an estimated 64 detainees and injuring 70 others. In late 2019, MSF witnessed a decrease in access to DC’s, as well as a decrease in the number of operational DC’s, as the conflict in Tripoli escalated.

MSF’s access to DCs is negotiated on a weekly basis, is unpredictable and does not always include direct access to all cells and detainees. Access has only been partially granted in 65% of the months that MSF has attempted access DCs. The number of detainees in our dataset represents a snapshot of those detainees to which MSF had access to on the last visit of the recording month; there is a high level of fluctuation from month to month. This fluctuation may relate to many factors, including detainees being transferred between different DCs, as well as migrants, refugees and asylum seekers being intercepted at sea and brought to DCs; additionally migrants, refugees and asylum seekers are often arrested during neighbourhood raids and then put under detention, and finally detainees may be evacuated, released or sold or are returned to their home countries [7, 8, 16, 17, 34]. This level of transfer makes monitoring and follow-up of patients extremely difficult. From one day to the next, people can be transferred between different DCs or moved to undisclosed locations. Some patients disappear without a trace.

The minimum standards for WSH in prisons as outlined by the International Committee of the Red Cross [31] and for humanitarian setting as outlined by SPHERE [32] were rarely met and “minimum conditions of human dignity” [35] are far from being reached. MSF documented severe overcrowding, especially for male detainees, in six out of eight DCs to which MSF had access. The minimum standard for living space of 3.5m^2^ per person, with adequate room for sleeping and daily activities and privacy [31, 32], was met at no time throughout the 18 months of observation, for five DCs for male detainees, and for three DCs for females detainees. Such overcrowding is known to increase the risk of person-to-person transmitted diseases [7, 17]. All DCs consistently provided insufficient ventilation, in relation to accepted minimum standards, which may increase risks for airborne infections such as TB and other respiratory infections. These WHS conditions in detention are corroborated by several agencies [7, 17, 25] and testimonies of individuals that were rescued by MSF on the Mediterranean Sea reporting living on minimal space, sometimes remaining in standing position for days or being detained in underground cells with no ventilation and minimal food and water in DCs in Tripoli. In our study, access to water and sanitation did not meet minimum standards [31, 32] in several DCs, despite MSF providing support to attempt reaching those standards. Even where minimum standards for availability of water for washing, toilets and showers were met, access to these facilities was sometimes restricted to certain hours of the day. Overnight access to water and toilets was not always possible for detainees. Lack of space, poorly planned facilities and access restrictions limited opportunities to improving water and sanitation, and facilitated the spread of water-washed (e.g. skin and eye infections) and water-borne diseases (e.g. faecal-orally transmitted diseases), and confirming information previously reported by the United Nations and non-governmental organisations [7, 17].

MSF conducted 25,328 OPD consultations in 12 months, for which 92.8% were for conditions presented for the first time. In several DCs, MSF carried out as many or more consultations for newly diagnosed diseases as there were detainees within the DC–potentially indicating an unusually high need for medical care among detainees. MSF conducted 953 SRH consultations among 2,942 women and first time ANC consultations among 4.9% of all women detainees—indicating that at least 4.9% of all women in detention were pregnant. MSF was consulted for 55 cases of sexual and gender-based violence, which probably represents a lower bound to the estimated number of incidents that may have taken place. Women ex-detainees repeatedly reported sexual abuse, and occasional sexual abuse of men was also reported in testimonies to MSF by ex-detainees rescued by MSF on the Mediterranean Sea. Additionally, MSF identified 190 newly diagnosed cases of TB among detainees. While it is not possible to calculate TB incidence rates, because of the lack of robust denominator data, with overall population numbers changing by an unknown extent each month, the numbers seen point to a likely incidence similar to those seen in high-burden TB countries [36]. TB in detention is likely under-detected due to lack of systematic screening, limited diagnostic tools and lack of access by medical staff. At the same time transmission risks are high, driven by poor living conditions and overcrowding in large communal cells with little daylight and poor ventilation.

We know of no comparable data pertaining to the health of detainees in Libya, and no other comparable settings to the one studied here, where migrants, refugees and asylum seekers from multiple countries of origins and migration routes are placed arbitrarily, and detained in overcrowded cells with little light and ventilation and often lack of water for an undetermined time period. The diseases that detainees presented with at OPD consultations speak to the living conditions and show hardly any seasonal variation, as would be typically expected for many diseases. Our findings are corroborated by results from studies about the health of migrants, refugees and asylum seekers arriving in Europe by boat–studies from Greece and Italy described journey- and living- condition-related illnesses such as respiratory tract infections and skin diseases such as scabies as the most common presentations [37–39]. Musculoskeletal diseases account for nearly one quarter of all diseases, year-round, conceivably linked with the lack of space in the cells and limited opportunities to leave cells, resulting in few or no possibilities to stretch and exercise, sometimes for several months. Acute upper and lower respiratory tract infections account for an additional quarter of all diseases, showing little typical seasonal variation and accounting for 14% of all diseases even in the month with the lowest proportion of respiratory tract infections (September 2019). Substandard ventilation and overcrowding may conceivably contribute to the spread of airborne diseases, driving conditions that result in continuous transmission of infections. Diseases potentially related to overcrowding, such as musculoskeletal conditions and respiratory tract infections, are followed in frequency by water-washed diseases. Water-washed diseases, that occur more frequently in settings with lack of water for personal hygiene—e.g. scabies, other skin diseases and eye infections—account for one sixth of all diseases and were also reported as very frequent diseases among migrants, refugees and asylum seekers in reception centres in Greece and Italy [37–39]. Diet-related diseases, e. g. heartburn, reflux and constipation, account for nearly one in eight diseases diagnosed; and corroborate observations from the MSF project team and other non-governmental organisations (NGOs) reporting a monotonous diet, provided at irregular intervals and in low quantities [7, 17]. We have additionally observed 274 cases of severe dehydration in DCs, which might be partially due to restricted access to drinking water and poor water quality, which was reported as salty by several ex-detainees in testimonies to MSF. 190 detainees were diagnosed with wounds and burns, linked with violence. In testimonies, ex-detainees reported physical abuse in DCs in Libya, often including beatings and electric shocks, however we did not have the means to verify the reports and the form of captivity. More than one sixth of all adults screened as part of the nutrition screening programme in one DC met criteria for malnutrition, based on BMI measurements. We have no direct robust data but do have access to anecdotal reports of lack of food and malnutrition in the DC’s [7, 17, 34]. We were able to show an association between lower MUAC and BMI measurements and increasing length of stay.

Less than one percent of consultations were among children aged under five years. With 45% of consultations among children less than five years for respiratory tract infections and 23% for skin infections, the adverse health effect of overcrowding seems to be even more pronounced in young children.

Overall, the poor living conditions and type and quantity of health problems our teams have documented, speak to unbearable conditions and as MSF we are faced with numerous ethical dilemmas [28]. MSF aims to put the interest of vulnerable groups at the centre of its decisions: we continue to provide health care and WHS support, while advocating for changes to migration deterrence practices [28–30], providing relevant data that substantiates our statements with numbers, and being transparent about the compromises we have to make.

### Limitations

All data presented was collected as routine MSF programme data and was not collected specifically for research purposes. Thus, some of the data, e.g. population numbers and WHS data is incomplete or inconclusive and could only partly be utilised in this analysis. While all procedures and case definitions stayed the same throughout the observation period, we cannot exclude that staff turnover and limitation in access may have led to variations in documentation and measurements. Furthermore, population numbers provide a point estimate for the population count on the last visit of the month, and cannot serve as a denominator for estimating incidence, as fluctuation in the population is not captured.

It is unclear whether the population observed is representative of the overall population of migrants, refugees and asylum seekers in detention in Tripoli. There is a possibility that the health of the overall population in detention is poorer than the health status of the cohort studied here, because detainees included in our analysis had access to MSF healthcare, which is not the case for all migrants, refugees and asylum seekers in detention in Tripoli and Libya.

MSF attempts to ensure maximum privacy during patient consultations. However, MSF is often required to carry out consultations with limited privacy and sometimes in the presence of guards. It is therefore likely that detainees with sensitive complaints might not access care or might not feel able to explicitly describe their problems. We therefore believe that problems linked with mental health concerns, or with exposure to physical or sexual and gender-based violence, are likely underreported in MSF consultation data [7, 17]. The fact that individuals who experienced detention in Libya and were subsequently interviewed on board of the MSF rescue ship on the Mediterranean Sea, reporting physical and sexual abuse in DCs, speaks to the incompleteness of our data in relation to these concerns.

Additionally, we did not conduct systematic screening for TB, hence our data may underestimate the true numbers of TB cases.

Furthermore, the linear regression model carried out using the nutrition screening data did only include age and self-reported duration of stay and no additional data on potential confounders and thus might under- or overestimate the effect of length of stay on MUAC and BMI. In addition, age and length of stay was self-reported and could not be verified. As we do not know how BMI and MUAC evolved over time among detainees, we cannot prove causality of length of stay and malnutrition but merely show a correlation that indicates that people who report longer length of stay display lower MUAC and BMI. Lastly, we do not know if the findings from the nutrition screening in one DC can be extrapolated to all DCs in Tripoli.

## Conclusion

Detention of men, women, children continues to take place in Tripoli. Our data come from a context within which it is not possible to guarantee access to healthcare, with MSFs’ access to DCs being renegotiated on a weekly basis. We describe a situation in which living conditions for detainees often fail to meet minimum requirements for water, sanitation and hygiene provision, as well as for living space and ventilation. Health problems that are diagnosed within MSF’s consultations reflect the conditions within which migrants, refugees and asylum seekers are held, and include diseases linked with overcrowding, lack of water and ventilation and poor diet. Every month that individuals remain in detention was found to be linked with an increased risk of malnutrition. Additionally, detainees presented to MSF with violence-induced wounds and reports of sexual and physical abuse.

The documented living conditions and health problems call for an end of detention and better protection of migrants, refugees and asylum seekers in Libya. This can only be achieved if pressure to enforce human rights of migrants, refugees and asylum seekers in Libya is increased, if Europe ends its support to the system of forced returns and subsequent detention of migrants, refugees and asylum seekers to Libya, and if safe and legal pathways out of Libya become available to those migrants, refugees and asylum seekers trying to flee the country and reach safety and a dignified existence.

Since late 2020, DCs in Tripoli are increasingly reopening as the wider conflict decreases and migrants continue to be forcibly returned back to Libya when intercepted by the Libyan Coast Guard at the Mediterranean Sea. However, detention of migrants, refugees and asylum seekers in Libya does not meet minimum conditions of human dignity and negatively impacts on detainees’ health.

## Abbreviations

ANC: antenatal care
BMI: body mass index
DC: detention centres
IPD: Inpatient department
MSF: Médecins Sans Frontières
MUAC: Mid-upper arm circumference
NGO: Non-governmental organisations
OPD: Outpatient department
PNC: Postnatal care
SGBV: Sexual and gender-based violence
SRH: Sexual and reproductive health
TB: tuberculosis
WHS: water, hygiene, and sanitation
